# Everybody's Page

**Published:** 1890-01-18

**Authors:** 


### Everybody's Page.

Two of the essentials of a good chemical disinfectant are that it should be poisonous and cheap.

The climate of California is found to be so favourable to the cultivation of the olive that orchards are being planted now on a large scale.

Economy is a delightful quality, and here is an example of it. The Chinese, after winding the silk from the silk-worm cocoon, eat the chrysalis.

Powdered starch, boric acid, and tincture of benzoin are recommended as a good snuff for coryza. They should be pounded together in equal parts, and then dried with gentle heat.

A large traffic in horse flesh is carried on between Newcastle-on-Tyne and Antwerp. It is stated that the horses are divided into two classes; the first, when slaughtered, being sold as beef, the second being made into sausages.

A sort of mummy is found in Australia, which has been prepared by fire and smoke; when the corpse is quite dried it is packed up into a bundle and carried about by a relative till nothing remains but the bones, and these are finally buried.

Notwithstanding that the water supply generally of Chinese towns is contaminated, their cities are remarkably free from diseases. This immunity is probably due, in no small degree, to the fact that the Chinese never drink cold water.

The number of hairs on the human head are said to vary according to their colour, the light coloured growing thicker than the coarse and dark coloured hair. According to estimates which have been made, about 850 hairs grow to the square inch in chestnut colour, 750 in flaxen hair, and 600 in black hair.

Eggs are said to become unwholesome when kept in refrigerators; a fungus forms in them which is easily found by the microscope, although it is not noticeable to the taste. This fungus constitutes a danger when we consider how many eggs are consumed by all classes of society, and people of delicate constitutions ought to be particularly careful that they eat fresh, and not kept, eggs.

Influenza can, it is said, be traced as far back as the year 412 B.C.; it appeared again in 591 as an epidemic all over Europe, and affected cattle as well as men, and when Pope Gregory instituted a grand procession through Rome 80 persons fell down dead. Some think that the old custom of saying to anybody who sneezes "God help thee," or "God bless thee," or "Your health" may date from the fatality at Rome. In the great outbreak, in 1782, 40,000 people were attacked in St. Petersburg.

"Hospitals," said Burke, "are lightning conductors to draw off the wrath of God from a sinful city, and give men an opportunity of expressing by action that feeling of compassion which sweetly urges them to succour the sick and afflicted, the most helpless of God's creatures," and everybody is rejoicing that "a feeling of compassion" has prompted a generous donor to give £100,000 for that most needful of hospital accessories, a convalescent home. Those who are used to being carefully nursed through their tedious convalescence, and who are given every sort of dainty nourishment, can feel for the people who often have to go back from the hospital, feeling weak and ill, to a noisy street and a noisy house full of children, and to badly cooked, unwholesome food.

The addition of a little gum-arabic or sperm salt improves boiled starch.

Some of the finest hospitals in the world are the municipal hospitals in Brazil. The Misericordia at Rio Janeiro receives 14,000 indoor patients yearly.

A little over one hundred years ago coffee was first cultivated in Brazil; that country now produces 60 per cent. of the coffee of the world.

The annual production of oleomargarine in the New England States is said to average about four million pounds. Rhode Island and Connecticut are responsible for the greater quantity of this.

Twenty-one years ago the Metropolitan Board of Works in London had only one park under its control; at the present time its successor has under its management over forty open-air places of recreation with an extent of nearly 3,000 acres.

It is always satisfactory to hear of new charitable institutions being opened which make no distinction of race or creed. The new Isabella Home, in New York, will accommodate 145 inmates, and report says it has cost 600,000 dollars.

One part of salicylic acid mixed in three parts of spermaceti ointment is said to be very efficacious for the relief and cure of corns. Fill up the hole in an ordinary corn plaister with this preparation, and put a piece of adhesive plaister on the top.

We hear so much nowadays of microbes, micro-organisms, and germs as agents in disease, that it is well to remember that many of the germs we daily breathe and swallow in such numbers are not only perfectly harmless, but are of benefit, as they live on their disease-producing brethren.

The *Kew Bulletin of Miscellaneous Information* gives some interesting information about the weather plant, the praises of which as a forecaster of weather and earthquakes were somewhat prematurely sung about eighteen months ago. The results of the experiments made by Dr. Oliver and Mr. Weiss show that the plant is quite unreliable as a prophet.

The *Journal de Medicine* gives a solution of the difficulty as to whether the epidemic is dengue or grippe. It says that to all enlightened minds it will not seem impossible that the microbes of dengue and grippe have met somewhere, perhaps in the steppes of Russia, and that the microbe of influenza is a combination of the two, and so there is a little of grippe and a little of dengue in this very unpleasant complaint. If, says the *Journal*, this very ingenious explanation does not reconcile everybody, it is because the actual epidemic has considerably obscured their intelligence. All we can say is that the combination is an uncommonly nasty one.

There are a great many people who still think that to wear warm clothing is to molly-coddle, and it seems almost impossible to make them believe that when we suggest they shall wear woollen clothing, it is not of necessity heavy and clumsy. Over and over again through lectures and papers and all sorts of channels this subject has been ventilated, and the same people who condemn what they consider the fuss that is made about wool nowadays, will cheerfully wear all manner of heavy cotton petticoats or let their feet freeze in cotton stockings, and refuse to believe that one light pure wool garment will go further to gain them comfort and health than any amount of cotton.

